# Discovering Multi-Scale Co-Occurrence Patterns of Asthma and Influenza with Oak Ridge Bio-Surveillance Toolkit

**DOI:** 10.3389/fpubh.2015.00182

**Published:** 2015-08-03

**Authors:** Arvind Ramanathan, Laura L. Pullum, Tanner C. Hobson, Christopher G. Stahl, Chad A. Steed, Shannon P. Quinn, Chakra S. Chennubhotla, Silvia Valkova

**Affiliations:** ^1^Computational Science and Engineering Division, Oak Ridge National Laboratory, Oak Ridge, TN, USA; ^2^Health Data Sciences Institute, Oak Ridge National Laboratory, Oak Ridge, TN, USA; ^3^Department of Computational and Systems Biology, University of Pittsburgh, Pittsburgh, PA, USA; ^4^IMS Health Government Solutions, Plymouth Meeting, PA, USA

**Keywords:** disease co-occurrence, non-negative matrix factorization, public health surveillance, asthma, flu, electronic healthcare reimbursement claims

## Abstract

We describe a data-driven unsupervised machine learning approach to extract geo-temporal co-occurrence patterns of asthma and the flu from large-scale electronic healthcare reimbursement claims (eHRC) datasets. Specifically, we examine the eHRC data from 2009 to 2010 pandemic H1N1 influenza season and analyze whether different geographic regions within the United States (US) showed an increase in co-occurrence patterns of the flu and asthma. Our analyses reveal that the temporal patterns extracted from the eHRC data show a distinct lag time between the peak incidence of the asthma and the flu. While the increased occurrence of asthma contributed to increased flu incidence during the pandemic, this co-occurrence is predominant for female patients. The geo-temporal patterns reveal that the co-occurrence of the flu and asthma are typically concentrated within the south-east US. Further, in agreement with previous studies, large urban areas (such as New York, Miami, and Los Angeles) exhibit co-occurrence patterns that suggest a peak incidence of asthma and flu significantly early in the spring and winter seasons. Together, our data-analytic approach, integrated within the Oak Ridge Bio-surveillance Toolkit platform, demonstrates how eHRC data can provide novel insights into co-occurring disease patterns.

## Introduction

The digitization of health records has spurred the systematic collection and archival of massive datasets, both within hospital and government computer systems (1–6). Therefore, digital public health surveillance is emerging as an important tool for tracking, monitoring, and driving decisions regarding emerging infectious disease spread within geographically distributed populations (7). Many bio-surveillance systems rely on the use of event-based, unstructured digital data, such as news feed aggregators, internet search patterns of users, and social media (7). However, with the availability of electronic health records (EHR) and electronic healthcare reimbursement claims (eHRC), there is a tremendous opportunity to seek, collect, monitor, and analyze these large-scale datasets for public health surveillance. While EHRs capture a patient’s full medical history, eHRCs capture only the healthcare reimbursements processed by insurance companies. In particular, eHRCs serve as a data warehouse that include claim transactions processed: (a) when patients visit their providers’ (e.g., doctor’s/nurse practitioner’s) office and/or (b) when retail pharmacies dispense prescription drugs to patients.

In this paper, we present a novel data-driven approach to extract co-occurring patterns of influenza-like illnesses (ILI) and asthma using eHRC datasets. While both conditions represent significant respiratory ailments, ILI occurs seasonally and asthma is a chronic condition that can result in wheezing, breathlessness, and cough. With an increasing number of patients being diagnosed with asthma since 2000 (8–10), we wanted to explore the relationship between the occurrence of asthma with the flu during the 2009–2010 pandemic flu season. In particular, the risks associated with influenza in young children susceptible to asthma have been well studied (11–14). However, the co-occurrence of flu and asthma in adults is less understood (15, 16). Therefore, we hypothesized that during the 2009–2010 H1N1 pandemic flu season, people who were more susceptible to asthma were likely to also be affected with the flu. Further, we hypothesized that the continued incidence of asthma within specific geographic regions in the US would predict which regions could be significantly affected by the flu.

To evaluate these hypotheses, we describe a novel unsupervised, machine learning approach to automatically identify spatial and temporal patterns from large-scale eHRC datasets for the 2009–2010 influenza and study its inter-relationship with asthma incidence during the same time period. Apart from discovering a small number of distinct geo-temporal patterns, our analysis shows a distinct lag in the temporal patterns of asthma and flu, i.e., we find that a peak in the number of diagnosed flu cases followed a peak in the number of diagnosed asthma cases. Our results further include an exploratory analysis into the demographic features of why such a peak may have been observed. In particular, we observe a behavior of the epidemic within large urban areas where environmental factors may have a significant impact (in addition to other factors) in influencing the total number of patients with flu and asthma.

## Materials and Methods

### Data

Prior to our study, we obtained internal Institutional Review Board approval for analyzing the IMS Health datasets. In this study, we analyzed the IMS Health ambulatory care reimbursement claims data from the 2009–2010 pandemic (H1N1) flu season; the details of the datasets are provided in our previous paper (17). Note that the data from IMS Health are already processed by a third party to remove any form of personally identifiable information before it receives the claims data from its suppliers. The study included eHRC from April 1, 2009 to March 31, 2010 with a total of nearly one billion records. We processed the ambulatory care reimbursement claims data and parsed out influenza (ICD9 codes 486XX and 488XX) and asthma (ICD9 codes 493XX) related records. We specifically chose those ICD9 codes that corresponded to hospital diagnosed cases of the flu. For flu, we obtained a total of over six million individual records (throughout the US). For asthma, we obtained a total of over 10 million individual records. We used the zip code corresponding to the patient’s service provider (i.e., a medical practitioner/physician), since the provider’s five-digit zip code is more specific than the patient’s three digit zip code directly accessible from the data. Only 0.0001% of the total records had different three-digit zip codes available for the patient and service provider.

The resulting flu and asthma datasets were stored as matrices, **A**_f_ and **A**_a_, respectively, where the rows represent the number of days and the columns represent the total number of zip codes. Note that the datasets are proprietary to IMS Health and therefore we cannot freely share the derived datasets used in this analysis. In order to characterize the co-occurrence of asthma and flu, we obtained a list of common zip codes between **A**_f_ and **A**_a_ and considered only those zip codes that had more than 10 reported cases of either diagnostic code set. The IMS diagnostic dataset covered 14,098 zip codes with statistically significant data for both flu and asthma, covering about 47% of the US.

### Identifying geo-temporal patterns using non-negative matrix factorization

Our primary hypothesis from the flu and asthma incidence patterns was to observe if the people susceptible to asthma were more likely to be infected with the flu, during the 2009–2010 H1N1 pandemic flu. We also wanted to understand if the continued presence of asthma within specific geographic regions would be predictive of the flu incidence in that area. To answer these questions, we used non-negative matrix factorization (NMF) to extract a small set of spatial and temporal patterns from the flu and asthma eHRCs. As we have shown in our previous paper (17), we chose NMF as an unsupervised machine learning technique to analyze the data primarily based on several empirical observations about the data. First, the data matrix **A** consists of only non-negative entries – because the total number of patients at any given zip code will be ≥0. Further, we observed that the individual zip codes exhibit a small number of distinct patterns in the occurrence of the flu (17), suggesting that in spite of a high dimensional setting of the **A**_asthma_ and **A**_flu_ matrices (with more than 14,000 individual zip codes and 365 days), there might be only a small number of geo-temporal patterns that could best capture the co-occurrence of these two conditions. Second, while several other types of analyses are possible to examine the data, our choice of analysis was motivated by the need to discover the underlying geo-temporal patterns in an unsupervised manner. Techniques, such as principal component analysis, which represents one of the most widely used unsupervised analysis technique, pursue variance blindly (18, 19) may fail to capture the intrinsic orientations in the high dimensional data space.

Given a data matrix **A**, with non-negative entries, *N_z_* × *N_t_* dimensions where *N_z_* represents the number of zip codes and *N_t_* represents time (in days), NMF finds low-rank approximations in *s* dimensions of the form **A** ≈ **WH**, where **W** with *N_z_* × *s* dimensions represents spatial patterns and **H** with *s* × *N_t_* captures temporal patterns within the data matrix. We used the alternate least squares algorithm proposed by Paatero (20, 21), available as part of the Matlab package. We ran NMF for a total of 1,000 iterations. To find the appropriate low-rank approximation (*s*), we varied *s* = 1, …, 15, dividing the original data into training and testing data (50% training and 50% testing). Tracking the residual errors using the Frobenius norm for both training and testing data, we performed a total of 250 iterations. In our analysis, as shown in previous work (17, 22), we identified *s* to be 5. Once we chose *s*, the most stable version of the basis matrices (**W**, **H**) by computing the Kullback–Leibler (KL) divergence between every pair of the 250 instances of **W** (or **H**) from the training set and picking **W** (or **H**) with the lowest KL divergence value.

## Results

### Flu and asthma case-counts during the 2009–2010 H1N1 pandemic season

**Figure 1:**
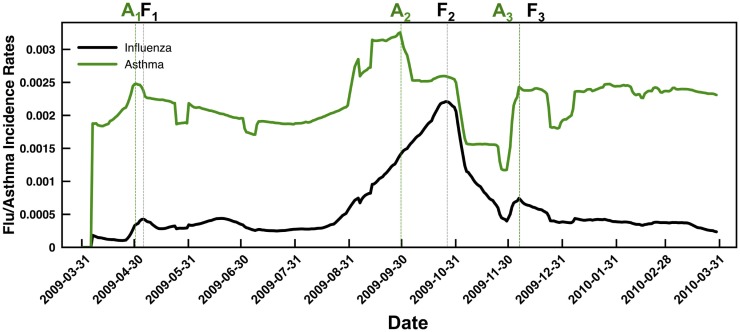
**Summary of temporal trends observed from the flu (black line) and asthma (green line) case counts indicate a distinct delay in the peak incidence of flu compared to asthma**. Note that we have reported the data using a moving average window of 7 days (to overcome gaps in the IMS ambulatory care reimbursement claims data based on reports received throughout the week) and normalized the results based on the fraction of total case counts received at every zip code. Dotted lines are used to the respective peak incidence rates of asthma (green) and flu (black). Note that in both the spring season (April–May 2009) and the fall season (September–October 2009), the asthma incidence (indicated by A**_1_** and A**_2_**, respectively) peaks before the peak in flu incidence (indicated by F**_1_** and F**_2_**, respectively). Only for the winter season, the peaks in asthma and flu incidence rates coincide (highlighted by A**_3_** and F**_3_** respectively).

We summarize the flu and asthma from the ambulatory care reimbursement claims data as a function of daily incidence. While influenza rates rise sharply during the August–September 2009 time-frame, the number of asthma cases observed from the data shows more or less a uniform distribution throughout the year, except for a slight increase and decrease around the time of the pandemic flu season. Further, we find that the peak number of asthma incidences lags behind by about 3 weeks when compared to the peak number of influenza incidences (Figure 1). Except for the beginning of the winter season in Figure 1, highlighted as **A**_3_ and **F**_3_, where the asthma and flu incidence rates coincide, in the other two cases, highlighted by **A**_1,2_ and **F**_1,2_, the peak incidence of asthma occurs earlier than the peak incidence of the flu. Note that for Figure 1, we present the data that were temporally averaged by 7 days (to account for lag times within diagnostic data reporting within the IMS Health datasets). We note that even without the temporal averaging, these trends are observed (both at state and national levels).

Once we examined the temporal trends in the flu and asthma datasets, we then extended the analysis to examine the demographic data for the total number of cases observed (both for H1N1 flu and asthma). Note that the IMS Health data include only information regarding the age of the patient and their sex, but do not include any other demographic information. As summarized in Table 1, we find that a larger proportion of children show increased co-occurrence of flu and asthma symptoms. Although the number of adults diagnosed with asthma is higher, only a small proportion of patients are co-diagnosed with both flu and asthma during the 2009–2010 flu season. Interestingly, in our analysis of the data, girls tend to exhibit a higher risk (over 90% of girls are susceptible to both flu and asthma in Table 1). Similarly, within adults co-diagnosed with the flu and asthma, female patients tended to be higher in ratio compared to men. Table 1 also indicates that the total number of diagnosed cases with both flu and asthma conditions is very infrequent and indicate that children seem to be at a greater risk than adults.

**Table 1:** **Demographic summary of H1N1 and asthma case count summary observed from eHRC data**.

Child attributes	Flu	Asthma	Flu and asthma
Mean age	7	7	
<1 year	109586	171117	6972
1–2 years	279806	663782	27206
3–5 years	466094	1109834	51155
>5 years	1241575	2870801	118951
Total	2097061	4815534	204284
No. (%) of girls	48	47	92

**Adult attributes**	**Flu case counts**	**Asthma case counts**	**Flu and asthma**

Mean age	42	51	
18–24 years	226075	616275	12543
25–30 years	181821	536832	8962
31–35 years	138167	530525	7565
36–40 years	144418	648801	8543
41–45 years	133488	741015	8762
46–50 years	131245	856720	9093
>50 years	393298	4080939	25450
Total	1348512	8011107	80918
No. (%) of females	60	69	70

An interesting question that arises from the above analysis is whether there are specific geographic regions within the US (or time windows), which exhibit a concurrent occurrence of the flu and asthma. We present an approach to discover such co-occurring patterns in the next section.

### Temporal patterns in flu and asthma incidence

#### Identifying Optimal Subspace and Cross Validation

The dimensionality of the data for each of the matrices (**A***_f,a_*) is *N_z_* × *N_t_* where *N_z_* represents the total number of zip codes (14,098) and *N_t_* represents the time points (365 days starting from April 1 2009 to March 31 2010). The subscripts *f* and *a* for the **A** matrix represent the two conditions examined, namely the flu and asthma, respectively. We hypothesized that the flu incidence patterns would be composed of discrete spatial and temporal patterns, given the geographic size and spread of the US. Further, given prior knowledge that there were distinct “peaks” associated with the 2009–2010 pandemic, it is reasonable to use techniques that could elucidate discrete, yet sparse spatial and temporal patterns from this high dimensional data. Additionally, the entries within each of these matrices are non-negative (i.e., it is not possible to obtain a negative count for the number of patients with the flu or asthma). For this purpose, we used non-negative matrix factorization (NMF), a technique that can extract low-rank approximations from the data.

Given a data matrix **A** with non-negative entries (*N_z_* × *N_t_* dimensions), NMF finds low-rank approximations of the form **A** ≈ **WH**, where **W** (*N_z_* × *s*) captures spatial patterns and **H** (*s* × *N_t_*) represents temporal patterns within the data. We used the alternate least squares algorithm proposed by Paatero (20, 21), available as part of standard Matlab (Mathworks, Inc.). Although the size of *A_a,f_* are quite large, we did not find the speed of convergence a significant problem. We used a stopping value of 1,000 as the maximum number of iterations. To identify the appropriate subspace (*s*) dimensions for the original data, we iterated over *s* = 1…15 for both matrices, dividing the data into random yet equal-sized training and testing data. We tracked the residual errors using Frobenius norm for both training and testing data. For each choice of *s*, we repeated this process 100 times. Using this procedure, we chose the optimal *s* = 5, based on the most stable version of the basis matrices by computing the Kullback–Leibler (KL) divergence between every pair of the 100 instances of **W** from the training dataset and picking the **W** with the lowest KL divergence value.

#### Distinct Break-out Patterns Govern Flu and Asthma Incidence

**Figure 2:**
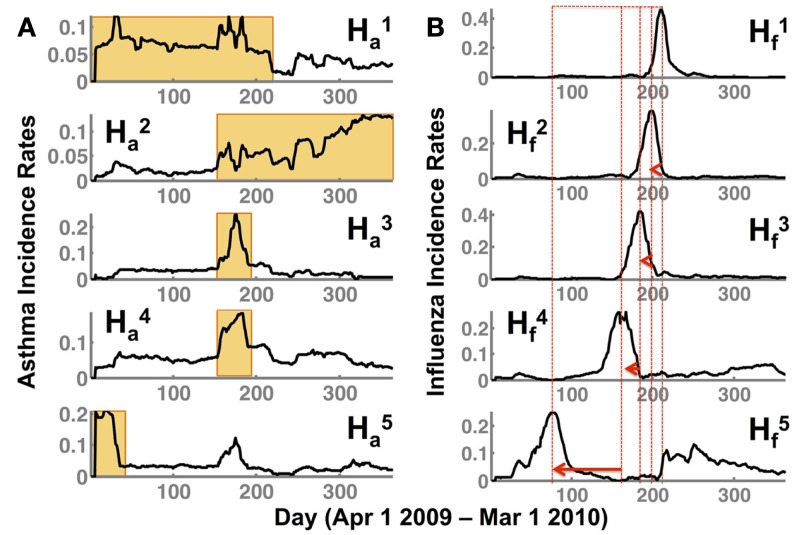
**NMF captures temporal patterns (H) in both (A) asthma (indicated by H*_a_*) and (B) flu (H*_f_*) showing a distinct lag time for the peak of the flu incidence**. The superscript indicates the respective subspace *s* from NMF that we are depicting. Note that the flu incidence rates have a distinct shift toward the spring/summer seasons (as indicated by the peak incidence rates in each of the patterns $H_{f}^{1,\ldots ,5}$, shown by a red dotted line and an arrow). While $H_{a}^{1}$ and $H_{a}^{2}$ indicate a sustained occurrence of asthma in the spring/summer and fall/winter seasons respectively, $H_{a}^{3}$ and $H_{a}^{4}$ indicate peak incidence of asthma preceding the flu incidence (corresponding to $H_{f}^{3,4}$). Further, the earlier onset of the flu observed in $H_{f}^{5}$ during the summer of 2009 is also preceded by a distinct peak in the asthma incidence observed in $H_{a}^{5}$.

NMF offers a convenient framework to interpret the incidence of flu and asthma throughout the US during the 2009–2010 time period. In particular, it provides a small number of basis vectors that describe temporal (**H***_f,a_*) and spatial (**W***_f,a_*) break-out patterns. Note that the subscripts used, *f* and *a*, correspond to the two conditions tracked, the flu and asthma, respectively. Based on the procedure outlined above, we selected the optimal subspace to be *s* = 5 as it sufficiently captured the underlying spatial and temporal patterns in the data, while providing an intuitive description how flu and asthma co-occurred at any given time period (or spatial location). As shown in Figure 2, one of the notable observations is that the temporal signatures are distinct in capturing the occurrence of flu and asthma in the 2009–2010 season.

The temporal patterns for asthma incidence, $H_{a}^{1}$ and $H_{a}^{2}$, show a seasonal rise in summer and winter season, respectively. In particular, the orange rectangles highlight the rise and sustained occurrence of asthma cases for the respective seasons. Interestingly, there is a very short overlap (of about 30 days from September to October) between the summer and winter where the asthma occurrence from one season overlaps with the other. This intersecting time period is captured as increased incidence rates in both $H_{a}^{3}$ and $H_{a}^{4}$. Additionally, in basis vector, $H_{a}^{5}$, we observe a high incidence of asthma around days 10–45 (April–May 2009) time-frame.

The break-out patterns for the flu across the US indicates at least three distinct peaks, ranging from days 180 to 210 (September–October 2009), 150 to 180 (August–September 2009), and 90–100 (June–July 2009). The temporal patterns from the flu data indicate that there is a distinct early onset of the epidemic ($H_{f}^{5}$), followed by several waves at later time-periods ($H_{f}^{1-4}$), which all have their own distinct temporal signatures. Thus, each of the basis vectors (in the flu dataset) captures a unique temporal break-out pattern that captures a different phase of the 2009–2010 flu epidemic, similar to previously reported studies in the spread of influenza (23).

Comparing the flu and asthma break-out patterns suggests that there is an overlap in the incidence of flu and asthma around August–September, described by $H_{a}^{3,5}$ and $H_{f}^{3,4}$, respectively. Further, comparing $H_{a}^{5}$ and $H_{f}^{5}$ also indicates that even during the early onset of the flu (days 90–100; June–July 2009), there is a marked increase in the asthma incidence rates around days 10–45 (April–May 2009). Although from Figure 1, we see that the overall trend indicates that the peak of asthma incidence precedes the peak of flu season, the analysis presented here further suggests that this precedence may be a distinct factor influencing the susceptibility of flu occurrence within some regions.

### Geographic patterns of flu and asthma incidence

**Figure 3:**
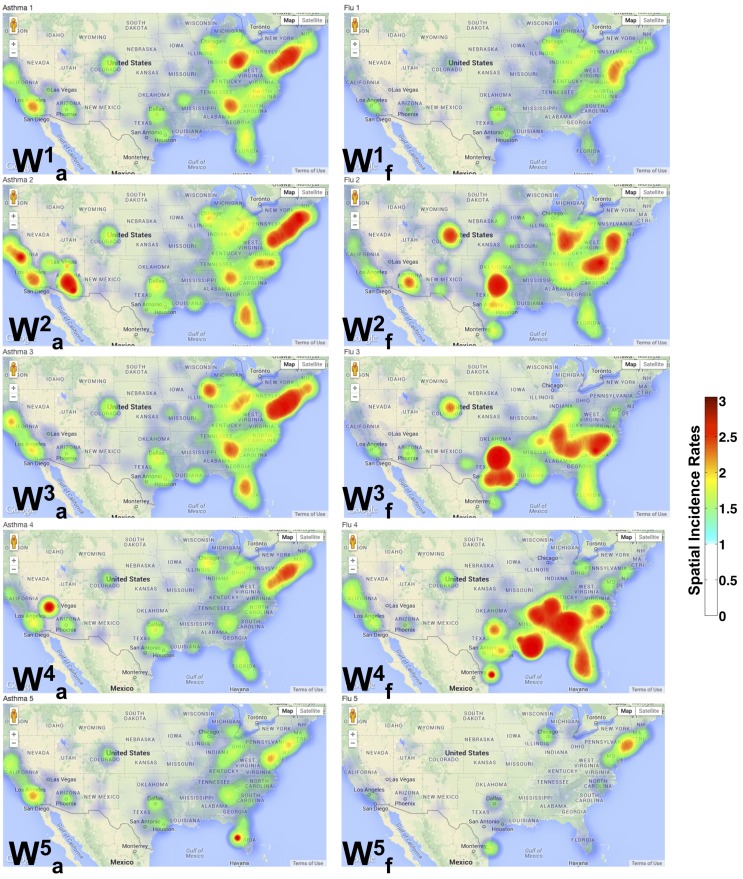
**Spatial patterns from NMF indicate distinct pockets of urban areas showing co-occurrence of flu and asthma**. A geographic incidence map of the flu ($W_{f}^{1,\ldots ,5}$) and asthma ($W_{a}^{1,\ldots ,5}$) shows the common areas of co-occurrence as described in our analysis of the diagnostic data. The spatial incidence is summarized in increasing color intensity shown on the color map. It is interesting to observe that the flu incidence gradually progresses to the south-east from $W_{f}^{1}$ through $W_{f}^{4}$. Further, $W_{f}^{5}$ almost exclusively describes the occurrence of the flu in only large urban areas of the country. Similar patterns are also observed in the asthma incidence with local incidences being concentrated around urban areas.

The spatial patterns summarized by NMF depict a distinct separation between the asthma and flu incidence. As shown in Figure 3, each **W** can be mapped onto the specific zip code and provides a geographic interpretation of the results presented above. Each dot represents a specific zip code examined, and the intensity of the color indicates a higher occurrence of the flu/asthma (blue indicates lower and red indicates higher incidence). Note that both the asthma and flu incidence maps are drawn to the same color scale (as indicated by the color bar in Figure 3).

We note that densely populated areas (such as New York, Florida, and California) constitute common grounds for the co-occurrence of the flu and asthma. In particular, throughout the north-east, southeast, west, and central US, asthma patterns are widespread. The spatial patterns for influenza across the entire US are, however, discrete. Several of the north-east states do not exhibit any patterns observed in $W_{f}^{2-4}$, meaning that during the latter half of the year, there were no many cases of the flu reported (except in urban areas). Within the urban areas of the north-east, however, the occurrence of asthma is quite widespread and occurs throughout the year.

The occurrence of $W_{f}^{4}$ is almost exclusive in the southern regions, with cases detected in both southeast and southwest (California). The temporal patterns from the south-east constitute the time-frame of August–September 2009, which signified the beginning of school season within the same region, leading to the unique spatial patterns observed here. Within the north-east (specifically in New York), the H1N1 pandemic was detected early (in April 2009) and a suitable warning was also issued (24), perhaps leading to a low number of observed flu cases during the peak time (September–October 2009) of the flu in these regions. The other interesting aspect observed from our analysis is the early onset of the flu in some north-eastern states (notably New York and New Jersey) as well as southwest (California), is captured by $W_{f}^{5}$, indicating that this early onset also meant a sustained flu in the later part of the season (around February–March 2010) in these regions (Figure 2B, $H_{f}^{5}$).

We also examined the zip codes where flu and asthma patterns occur concurrently. These regions include urban areas within the north-east (specifically, southeastern New York, New Jersey, Delaware, southern parts of New Hampshire, Connecticut, and Pennsylvania), southeast (Tennessee, Georgia, North and South Carolinas, Florida, and south central parts of Virginia), and the west-coast area (California, Oregon, and Washington states). These urban areas constitute a majority of the places where the co-occurrence of the flu and asthma exhibit a clear trend, i.e., a peak in asthma diagnoses is subsequently followed by a peak in the flu diagnoses. This is in agreement with previous studies that have showed that air quality, local weather, and pollen fluctuations, as well as presence of environmental pollutants within urban areas can significantly impact patients with asthma (25).

## Discussion and Conclusion

### Comparison with previous work

The use of EHR and eHRC datasets for bio-surveillance is relatively new (26). Privacy and security concerns within EHR and eHRC systems have made it tremendously challenging to engage local and public health departments in effectively collecting, sharing, and disseminating bio-surveillance-related data (27). Although eHRC transaction datasets have been routinely used in the context of tracking and analyzing pharmacy prescriptions and understanding drug efficacy [e.g., Ref. (28–32)], very little research has been carried out in terms of using them as potential data sources for digital public health surveillance. eHRCs have a distinct lag time associated with claims processing and being made available for analysis. Therefore, the timeliness of their availability from the claims processor may have a significant impact on assessing the data real-time, i.e., as epidemics are spreading through the population. A recent study showed that retail pharmacy sales data can be used as a reliable measure for syndromic surveillance; specifically, the aggregate counts of prescription sales of four anti-viral drugs for influenza correlated well with Google Flu Trends (33, 34). However, given the concerns with Google Flu (35), there is a need to develop alternate strategies to evaluate eHRCs in tracking flu (and other diseases). It should be noted here that these papers make use of standard time-series algorithms and/or other signal processing techniques to model the temporal trends and report correlations with existing and available CDC ILINet datasets.

Of the many approaches used to analyze bio-surveillance-related datasets, supervised and unsupervised machine learning techniques have been made use of in classifying text messages from various social media sources (such as Twitter) (36, 37). In addition, these techniques are used to rank search results of various bio-surveillance terms (from either a pool of Twitter documents or other internet-based surveillance sources) (38) to aid analysts in identifying the most relevant documents for decision making. However, these techniques have not been used in the context to identify co-occurring disease patterns for bio-surveillance.

In our previous work, we showed how diagnostic eHRC transactions are comparable to standard public health surveillance data, such as the CDC ILINet (17). Further, we also showed that the consolidated eHRCs at local (zip code level information), regional (county, metropolitan, city, state, etc.), and national levels can be used to assess how infectious diseases like the flu may spread. Unlike aggregating web-based search patterns by users (33, 35), or the use of social media (39–42), where the use of such data can significantly overestimate the flu incidence patterns (43, 44), using eHRCs provides a more accurate indication and potential predictors. To our knowledge, this study is perhaps the first to use eHRCs for identifying co-occurrence patterns of flu and asthma at the national scale.

Another body of literature examines how asthma and the flu represent syndemic conditions, meaning that both afflictions are linked and interact synergistically contributing to an excess burden of disease (14, 25, 45, 46). In this context, the mechanisms by which influenza can exacerbate asthma in patients have been well documented (47, 48). Studies have examined clinical strategies to vaccinate patients (both children and adults) against influenza that have asthma so that adverse reactions can be prevented (11, 15, 49–52) and also evaluated the general safety of the influenza vaccines and other treatments (such as anti-viral drugs) for patients with asthma (53, 54). Although our study did not examine whether the flu and asthma are syndemic within particular patient populations, we showed that the girls and, in general, female patients were more susceptible to be co-diagnosed with the flu and asthma during the 2009 H1N1 pandemic season. We believe that further analysis would be necessary, including the use of prescription eHRC datasets to glean whether treatments, such as anti-viral medicines or vaccinations for these susceptible patient sub-populations, were effective in controlling the pandemic spread.

From the analysis of the diagnostic data, we showed that it is possible to summarize the spatial and temporal patterns from these two conditions into a small number of categorical dimensions, each showing a distinct (temporal and spatial) signature with respect to the occurrence of asthma and flu. By examining the demographics of flu and asthma occurrence in both children and adult populations, we observed that a major proportion of girls and women were more susceptible to their co-occurrence. While it is widely acknowledged that older women are more susceptible to asthma in later ages (25), the co-occurrence of asthma and flu within younger females correlates well on the statistics in recent years, showing a higher percentage of girls affected with asthma attacks (10). Further analysis into the nature of incidence and reports would be needed, and we propose to examine this as part of future publications.

### Perspective and potential limitations

The analysis of the spatial patterns for flu and asthma revealed that there are distinct geographic locations (albeit a very small number of them, about 4,000/14,000 zip codes) that show more than one temporal signatures in the flu/asthma incidence patterns. Further analysis of these regions will be necessary to understand the origins of such “mixing.” In particular, as part of our analysis, we did not examine patient age or history to understand how a specific group of patients (or a demographic) may be more susceptible to asthma or the flu. Patients with one or more pre-existing respiratory conditions can be more susceptible to either flu or asthma and hence these factors would have to be taken into account to further understand the co-occurrence patterns observed during the 2009–2010 flu season. At the time of writing this paper, this information was not available.

We note here that a more detailed analysis of the spatio-temporal patterns is required. In particular, for this paper, we have not quantitatively examined how these temporal patterns match up against other known temporal mining algorithms and even other unsupervised machine learning techniques, such as principal component analysis. We also note that the predictive aspects of our algorithm have also not been fully explored for two reasons: (1) the data available to us are only from the 2009–2010 flu season and (2) it is difficult to obtain a baseline behavior based on a year that showed highly anomalous behavior in terms of the overall flu incidence across the entire country. We will explore these questions in greater detail in a following publication. Another potential limitation of our study is that we chose to aggregate our data based on individual zip codes. While the use of other aggregation techniques (e.g., HHS regions, or state-level) are more appropriate for epidemiological purposes, our goal within this study was to demonstrate how we can extract constituent patterns of asthma/flu incidence and observe correlated behaviors at this spatial resolution. We propose to examine standard approaches of epidemiological data aggregation in further studies.

The analytic techniques outlined here are part of the data analytic platform for public health surveillance that we have been developing (22). The platform was designed specifically to bring together heterogeneous datasets, such as social media and eHRCs, and analyze these datasets to gather insights into emerging public health concerns. In this study, we used asthma and influenza as specific examples to understand co-occurrence patterns across the US. However, the techniques are quite general and can be integrated with visual analytic tools to summarize, navigate, and interpret large volumes of complex healthcare datasets. We believe that the availability of unique datasets and data analyses techniques outlined above can lead to better public health surveillance systems and have a positive impact on the nation’s health.

#### Source Code and Availability

The Oak Ridge Bio-surveillance Toolkit (ORBiT) (17, 22) and the tools implemented as part of this paper will be made available as a open source Python-based package from our website (http://cda.ornl.gov). Data (used as part of this paper) can be requested through IMS Health Institute.

## Author Contributions

AR and LP conceived and designed the study. SV provided the data. TH and LP processed and stored the eHRC datasets for analysis. AR, LP, SQ, and CC developed the analysis techniques. TH, CAS, and CGS developed the user interface components for visualizing the results. AR, CC, SQ, and TH analyzed the data. AR, LP, TH, CGS, CAS, SQ, SV, and CC wrote the paper.

## Conflict of Interest Statement

The authors declare that the research was conducted in the absence of any commercial or financial relationships that could be construed as a potential conflict of interest.
